# LncRNA UCA1 promotes proliferation and cisplatin resistance of oral squamous cell carcinoma by sunppressing miR‐184 expression

**DOI:** 10.1002/cam4.1253

**Published:** 2017-11-10

**Authors:** Zheng Fang, Junfang Zhao, Weihong Xie, Qiang Sun, Haibin Wang, Bin Qiao

**Affiliations:** ^1^ Department of Stomatology the First Affiliated Hospital of Zhengzhou University Zhengzhou 450052 China

**Keywords:** CDDP resistance, lncRNA UCA1, miR‐184, oral squamous cell carcinoma, SF1

## Abstract

Chemotherapy resistance has become the main obstacle for the effective treatment of human cancers. Long non‐coding RNA urothelial cancer associated 1 (UCA1) is generally regarded as an oncogene in some cancers. However, the function and molecular mechanism of UCA1 implicated in cisplatin (CDDP) chemoresistance of oral squamous cell carcinoma (OSCC) is still not fully established. UCA1 expression in tumor tissues and cells was tested by qRT‐PCR. MTT, flow cytometry and caspase‐3 activity analysis were explored to evaluate the CDDP sensitivity in OSCC cells. Western blot analysis was used to measure BCL2, Bax and SF1 protein expression. Luciferase reporter assay was conducted to investigate the molecular relationship between UCA1, miR‐184, and SF1. Nude mice model was used to confirm the functional role of UCA1 in CDDP resistance in vivo. UCA1 expression was upregulated in OSCC tissues, cell lines, and CDDP resistant OSCC cells. Function analysis revealed that UCA1 facilitated proliferation, enhanced CDDP chemoresistance, and suppressed apoptosis in OSCC cells. Mechanisms investigation indicated that UCA1 could interact with miR‐184 to repress its expression. Rescue experiments suggested that downregulation of miR‐184 partly reversed the tumor suppression effect and CDDP chemosensitivity of UCA1 knockdown in CDDP‐resistant OSCC cells. Moreover, UCA1 could perform as a miR‐184 sponge to modulate SF1 expression. The OSCC nude mice model experiments demonstrated that depletion of UCA1 further boosted CDDP‐mediated repression effect on tumor growth. UCA1 accelerated proliferation, increased CDDP chemoresistance and restrained apoptosis partly through modulating SF1 via sponging miR‐184 in OSCC cells, suggesting that targeting UCA1 may be a potential therapeutic strategy for OSCC patients

## Introduction

Oral squamous cell carcinoma (OSCC) is one of the most common head and neck malignancies, occupying approximately 3% in all recently diagnosed clinical cancer cases 1. Although lots of critical progress has been made in recent years, the overall 5‐year survival rate of OSCC patients remain unsatisfactory and less than 50% 2. Chemotherapy is an efficient adjuvant treatment for OSCC patients in some cases. However, the emergence and development of resistance to chemotherapy drugs hampered the curative effect to a large extent 3. Cisplatin (CDDP) is a platinum‐based anti‐cancer drug used for a broad range of cancers, whereas, the severe side effect and generated resistance often limit its clinical application 4. Therefore, the better understanding of molecular mechanisms underlying CDDP chemoresistance acquisition in OSCC is essential and urgent for improving the therapeutic outcome of OSCC patients.

Long non‐coding RNAs (lncRNAs), a kind of transcript with over 200 nucleotides (nt) in length and without protein‐coding potential, have been shown as vital regulators in various gene expression and biological processes 5. Emerging evidence manifests that lncRNAs are implicated in the progress of multiple cancers at epigenetic, transcriptional, post‐transcriptional, and translational level 6. More importantly, lncRNAs‐mediated chemoresistance has been widely discussed in a great number of researches 7, 8. Urothelial cancer associated 1 (UCA1), initially discovered in bladder cancer and located at chromosome 19p13.12, contributes to cancer development via regulating cell proliferation, apoptosis, migration, and invasion in diverse tumors, such as breast cancer, colorectal cancer, tongue squamous cell carcinoma (TSCC), and so on 9. Studies also showed that the expression level of UCA1 in OSCC was strikingly upregulated and UCA1 exerted an oncogenic effect in the progress of OSCC 10. Moreover, the involvement of UCA1 in drug resistance was also disclosed in a variety of cancers. For instance, UCA1 promoted cell proliferation and conferred 5‐fluorouracil resistance in colorectal cancer 11. Decreased expression of UCA1 enhanced CDDP‐induced apoptosis and chemosensitivity in TSCC cells 12. It is indicated that lncRNA could act as competing endogenous RNAs (ceRNAs) to affect the expression of miRNAs, leading to alteration of target mRNAs expression 13. However, the molecular mechanism of UCA1 implicated in OSCC progression and CDDP chemoresistance is still not fully established. In this study, we aimed to investigate roles and molecular mechanisms of UCA1 in the progression and CDDP chemoresistance of OSCC.

## Materials and Methods

### Patient tissue samples and cell culture

OSCC tumor tissues and their corresponding normal tissues were achieved from 30 cases of patients diagnosed with OSCC at our hospital. Our study was approved by Research Scientific Ethics Committee of the First Affiliated Hospital of Zhengzhou University. All participants signed informed consent prior to using the tissues for scientific research.

OSCC cell lines (Tca8113, TSCCA, CAL‐27, SCC‐9) and normal human oral keratinocyte (NHOK) were all obtained from the Cell Bank of Type Culture Collection of Chinese Academy of Sciences (Shanghai, China). CDDP‐resistant OSCC cells (Tca8113‐CDDP and TSCCA‐CDDP), derived from CDDP‐sensitive cell lines Tca8113 and TSCCA, were established referring to the previous document 14. Briefly, the Tca8113 and TSCCA cells were treated with gradually increasing doses of CDDP until the survival cells exhibited a normal growth pattern. All cells were maintained in DMEM medium supplemented with 10% FBS (Invitrogen, Carlsbad, CA, USA) and cultured in a humidified air atmosphere with 5% CO_2_ at 37°C.

### Cell transfection

To construct UCA1 overexpression plasmid, full length of UCA1 cDNA sequence was amplified, cloned into pcDNA3.1 vector (Invitrogen) and sequenced, named as pcDNA3.1‐UCA1 (UCA1). The specific small interference RNA (siRNA) targeting UCA1 (si‐UCA1) and scrambled siRNA control (si‐NC) were obtained from GenePharma Co., Ltd (Shanghai, China). miR‐184 mimic (miR‐184), scrambled mimic control (miR‐NC), miR‐184 inhibitor (anti‐miR‐184), and inhibitor control (anti‐miR‐NC) were purchased from RiboBio (Guangzhou, China).All these plasmids and oligonucleotides were transfected into cells by lipofectamine 2000 reagent (Invitrogen) following the manufacturer's instructions.

### Generation of UCA1 stably knockdown cell line

The designed shRNA oligo targeting UCA1 and control oligo were cloned into pLKO.1 vectors to form pLKO.1‐sh‐UCA1 or pLKO.1‐sh‐NC plasmid, followed by co‐transfected with pspAX2 and pMD2.G into 293T cells to produce sh‐UCA1 or sh‐NC lentivirus. Constructed sh‐UCA1 or sh‐NC lentivirus was respectively infected into Tca8113 cells, which were then screened with puromycin for over 7 days.

### RNA isolation and RT‐qPCR assay

Total RNA was extracted and collected from OSCC tissues or cells using the TRIzol reagent (Invitrogen) referring the instructions of manufacturer. First‐strand cDNA was synthesized from 1 *μ*g of total RNA by miScript reverse transcription kit (Qiagen, Dusseldorf, Germany). The expression levels of UCA1 and miR‐184 were quantified by miScript SYBR‐Green PCR kit (Qiagen). The relative fold change for gene expression was calculated using 2^−ΔΔCT^ method, with GAPDH or U6 snRNA as internal control.

### Cell proliferation assays and drug sensitivity determination

OSCC cell proliferation was measured at the indicated time point (0, 24, 48, and 72 h) after transfection by XTT assays using Cell Proliferation Kit II (RocheMolecular Biochemicals, Mannheim, Germany). For drug sensitivity analysis, transfected OSCC cells were exposed to different concentrations of CDDP ranging from 0 to 160 *μ*mol/L for 48 h prior to XTT assays. Half maximal inhibitory concentration (IC^50^) was calculated with GraphPad Prism Version 5.0 Software.

### Dual luciferase reporter assays

Partial UCA1 sequences or SF1 3′ UTR fragments containing corresponding wild‐type or mutant‐type miR‐184 binding site were amplified by PCR and then subcloned into psiCHECK‐2 luciferase reporter vector (Promega, Madison, WI, USA), producing WT‐UCA1, MUT‐UCA1, WT‐SF1‐3′ UTR, and MUT‐SF1‐3′ UTR reporter plasmids. Then the reporter plasmids were co‐transfected with miR‐NC or miR‐184 into 293T cells. The luciferase activities in cell lysates were detected 48 h after transfection by dual luciferase reporter assay kit (Promega) referring the protocols of manufacturer.

### Western blot assays

The whole protein was extracted by pre‐cold RIPA buffer (Beyotime, Shanghai, China) with protease inhibitor, followed by separated on SDS‐PAGE and shifted to polyvinylidene fluoride membranes. After blocked in 5% nonfat milk for 1 h, the membranes were gently incubated overnight at 4°C with appropriate concentration of antibodies, including SF1, Bax, BCL2, and *β*‐actin (Santa Cruz Biotechnology, Dallas, TX, USA). Subsequently, the horseradish peroxidase (HRP)‐conjugated secondary antibody was employed to combine the primary antibody on the membranes for 1 h at room temperature. Protein bands were detected with the EasySee Western Blot Kit (Transgen Biotech, Beijing, China) and analyzed with Quantity One software (Bio‐Rad Laboratories, Hercules, CA, USA). *β*‐actin acted as an internal reference to normalize the expression of SF1, Bax, and BCL2.

### Cell apoptosis detection

CDDP‐resistant OSCC cells with different transfection were treated with 5 *μ*M CDDP for 48 h, followed by double‐stained with AnnexinV‐FITC/PI Apoptosis Detection Kit (Beyotime) according to the manufacturer's instructions. Cell apoptotic rates were assessed by FACSan flow cytometry (BD Biosciences, San Jose, CA, USA).

### Caspase 3 activity assay

The caspase 3 activity in OSCC cells was measured 48 h after transfection by Caspase 3 Activity Assay Kit (Beyotime) following the manufacturer's protocols.

### Xenograft mice model of OSCC tumor growth

The experiments were performed according to the protocol of the institutional ethics board of the First Affiliated Hospital of Zhengzhou University. Every effort was made to minimize the pain of mice. BALB/c nude mice (6 weeks old) were obtained from Shanghai SLRC Experimental Animal Center (Shanghai, China) and bred in specific conditions. Tca8113‐CDDP cells (1 × 10^7^) infected with sh‐UCA1 or sh‐NC were suspended in 100 *μ*L medium and then subcutaneously injected into the right flanks of mice. After 7 days, the mice began to receive an intraperitoneal administration of cisplatin (4 mg/kg) or an equal volume of PBS once every 4 days. At the indicated time points (7, 11, 15, 19, 23, 27, and 31 days) after inoculation, tumor growth was measured with a digital caliper. At the end of the experiment, mice were euthanatized for tumor weight analysis.

### Statistical analyses

All results were presented as mean ± standard deviation (SD) from at least three separate assays. Student's *t*‐test or one‐way analysis of variance (ANOVA) was used to compare the difference between two or more groups. *P*‐value of < 0.05 was considered as statistically significant.

## Results

### UCA1 expression was upregulated in OSCC tissue specimens, cell lines, and CDDP‐resistant OSCC cells

**Figure 1:**
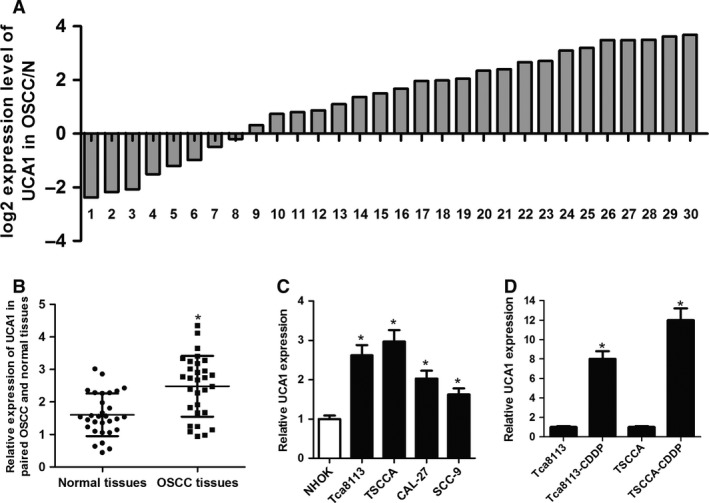
UCA1 expression was upregulated in OSCC tumor samples and cell lines. (A and B) Analysis of UCA1 expression levels in tumor tissues and corresponding noncancerous tissues of 30 OSCC patients. (C) Expression of UCA1 in normal human oral keratinocyte cell line (NHOK) and OSCC cell lines (Tca8113, TSCCA, CAL‐27, SCC‐9). (D) Expression analysis of UCA1 in OSCC cell lines (Tca8113, TSCCA) and their corresponding CDDP‐resistant cell lines (Tca8113‐CDDP, TSCCA‐CDDP). *P <* *0.05.

In order to investigate the effect of UCA1 in OSCC, RT‐qPCR assays were carried out to assess the respective expression pattern of UCA1 in 30 cases of OSCC tumor tissues and their corresponding adjacent normal tissues specimens. The results showed that the expression level of UCA1 was remarkably increased in 22 of 30 OSCC patients (73.3%) tumor tissues compared with their normal counterparts (Fig. 1A). The overall expression status of UCA1 indicated that UCA1 expression was higher in OSCC tissue specimens than that in pair‐matched normal tissues (Fig. 1B). Next, the expression levels of UCA1 in normal human oral keratinocyte cell line (NHOK) and OSCC cell lines (Tca8113, TSCCA, CAL‐27, SCC‐9) were also detected by RT‐qPCR. The outcome showed UCA1 expression was strikingly upregulated in all OSCC cell lines when compared to NHOK cells (Fig. 1C). To explore the involvement of UCA1 in the resistance of OSCC to CDDP, UCA1 expression in CDDP‐resistant Tca8113 cell (Tca8113‐CDDP) and CDDP‐resistant TSCCA cell (TSCCA‐CDDP) were detected. As presented in Figure 1D, CDDP‐resistant OSCC cells exhibited significantly higher expression of UCA1 than corresponding parental cells. These results indicated that dysregulation of UCA1 may be associated with the progression and CDDP resistance of OSCC.

### UCA1 promoted proliferation and inhibited CDDP susceptibility of OSCC cells

**Figure 2:**
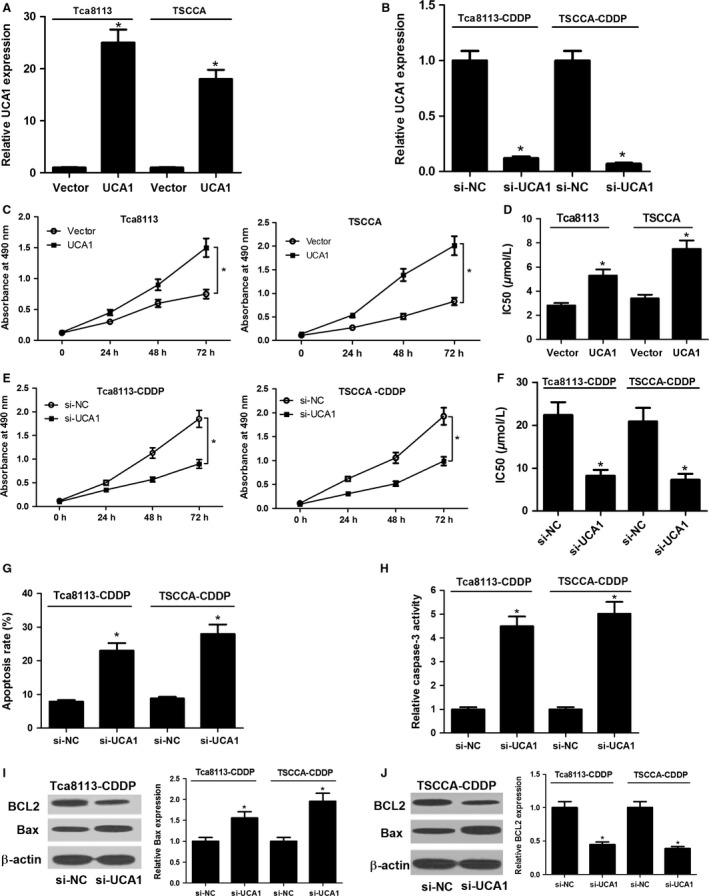
UCA1 facilitated proliferation and CDDP resistance of OSCC cells. (A and B) RT‐qPCR assays were used to confirm the efficiency of overexpression plasmid and si‐RNA of UCA1. (C) Effect of UCA1 overexpression on OSCC cell proliferation was measured by XTT assays. (D) Effect of UCA1 upregulation on CDDP resistance was determined by calculating IC_50_ value using XTT assays. (E) Effect of UCA1 knockdown on CDDP‐resistant OSCC cell proliferation was detected by XTT analysis. (F) Effect of UCA1 deficiency on CDDP resistance was assessed by computing IC_50_ value using XTT experiments. Tca8113‐CDDP and TSCCA‐CDDP cells introduced with si‐UCA1 or si‐NC were treated with 5 *μ*mol/L CDDP for 48 h. Effect of si‐UCA1 on cell apoptosis was detected by analyzing apoptotic rate (G), caspase‐3 activity (H), as well as Bax/BCL2 expression (I and J). *P <* *0.05.

To investigate the role of UCA1 in Tca8113 and TSCCA cells, the overexpression plasmid (pcDNA‐UCA1) and siRNA (si‐UCA1) of UCA1 were constructed or synthesized. As shown in Figure 2A, the expression levels of UCA1 in Tca8113 and TSCCA cells were dramatically upregulated following pcDNA‐UCA1 transfection compared to corresponding empty vector groups. Conversely, introduction of si‐UCA1 in the Tca8113‐CDDP and TSCCA‐CDDP cells obviously reduced the expression of UCA1 compared with the negative control groups (Fig. 2B). These results indicated that overexpression and knocking down systems of UCA1 were effective to increase or inhibit the expression of UCA1. Then the effect of UCA1 on cell proliferation rate was evaluated by XTT assays. As shown in Figure 2C, OSCC cell growth was markedly improved after UCA1 overexpression. Moreover, ectopic expression of UCA1 resulted in an increase of CDDP resistance in OSCC cells, presented by higher IC_50_ value (Fig. 2D). As expected, introduction of si‐UCA1 notably suppressed the OSCC cell proliferation (Fig. 2E). Moreover, UCA1 knocking‐down by si‐UCA1 prominently enhanced OSCC cell susceptibility to CDDP (Fig. 2F). In a word, UCA1 promoted proliferation and conferred CDDP resistance of OSCC cells.

### Downregulation of UCA1 promoted CDDP‐induced apoptosis in CDDP‐resistant OSCC cells

As mentioned above, si‐UCA1 transfection notably decreased the viability of Tca8113‐CDDP and TSCCA‐CDDP cells. Therefore, we attempted to investigate whether the growth inhibition was caused by the increase of apoptotic rate. Tca8113‐CDDP and TSCCA‐CDDP cells with the transfection of si‐NC or si‐UCA1 were treated with 5 *μ*mol/L CDDP for 48 h, followed by AnnexinV‐FITC/PI double staining and flow cytometry analysis. The results revealed that introduction of si‐UCA1 significantly increased CDDP‐induced apoptosis in CDDP‐resistant OSCC cells (Fig. 2G). To further validate the conclusion, the effect of si‐UCA1 on the expression of apoptosis‐related genes (caspase‐3, Bax, and BCL2) were also detected in CDDP‐resistant OSCC cells after treatment with CDDP. As displayed in Figure 2H–I, knocking down of UCA1 evidently upregulated caspase‐3 activity and protein expression of pro‐apoptosis gene Bax compared with negative control (si‐NC) group. Conversely, the protein expression levels of anti‐apoptosis gene BCL2 were downregulated in the presence of si‐UCA1 (Fig. 2J). In conclusion, silencing UCA1 by si‐UCA1 enhanced CDDP‐induced apoptosis in CDDP‐resistant OSCC cells.

### UCA1 inhibited miR‐184 expression

**Figure 3:**
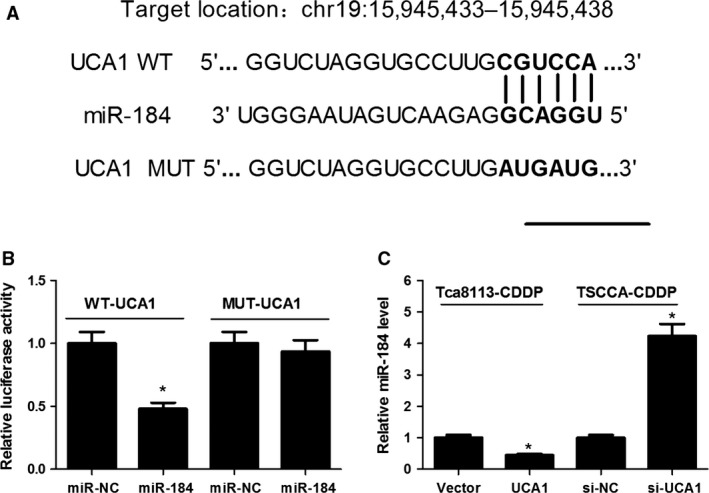
UCA1 interacted with miR‐184 to suppress its expression. (A) The putative binding sites and corresponding mutant region for UCA1 within miR‐184. (B) Effect of miR‐184 on the luciferase activity of WT‐UCA1 and MUT‐UCA1 reporter systems was measured by luciferase reporter assay in 293T cells. (C) Effect of UCA1 overexpression or knocking‐down on the expression of miR‐184 was respectively detected in Tca8113‐CDDP and TSCCA‐CDDP cells by RT‐qPCR. *P <* *0.05.

**Figure 4:**
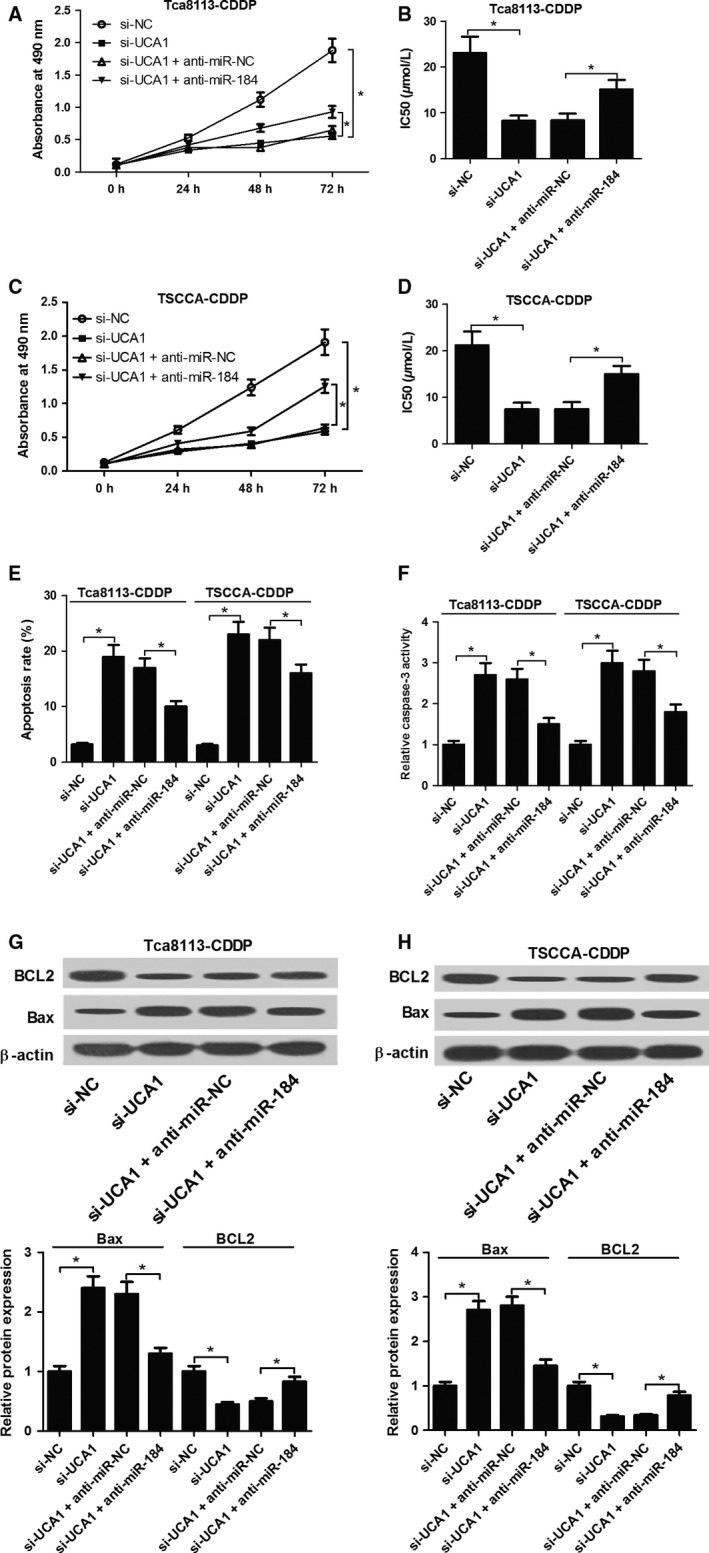
miR‐184 suppresion abated the effect of UCA1 knockdown on proliferation, CDDP chemosensitivity, and apoptosis in CDDP‐resistant OSCC cells. Tca8113‐CDDP and TSCCA‐CDDP cells were transfected with si‐UCA1 or in combination with anti‐miR‐184. (A and C) XTT assays were used to detect cell growth. (B and D) XTT assays were employed to evaluate CDDP sensitivity by calculating IC_50_ value. (E) Flow cytometry analysis of cell apoptosis. (F) Caspase‐3 activity analysis. (G and H) Western blot assays of Bax and BCL2 expression. *P <* *0.05.

Up to now, accumulating evidence indicated that lncRNAs exerted the function by interacting with miRNAs. Therefore, to uncover potential target miRNAs of UCA1, the bioinformatics predicton analysis was performed by miRcode online website. As shown in Figure 3A, miR‐184 harbor complementary binding sequence of UCA1 (Fig. 4A). In order to further validate the interaction, UCA1 sequence containing the putative or mutated miR‐184 binding site was cloned into the downstream of luciferase reporter gene, generating WT‐UCA1 or MUT‐UCA1 luciferase reporter plasmids. Then the effect of miR‐184 on WT‐UCA1 or MUT‐UCA1 luciferase reporter systems was determined. As displayed in Figure 3B, compared with miR‐NC, the luciferase activity of WT‐UCA1 reporter systems was apparently suppressed after 293T cells were co‐transfected with miR‐184 mimic. Whereas, introduction of miR‐184 mimic had no effect on the luciferase activity of MUT‐UCA1 reporter systems. These outcomes indicated that the interaction of UCA1 and miR‐184 was realized by the putative binding site. Subsequently, the effect of UCA1 on miR‐184 expression was also observed in Tca8113‐CDDP and TSCCA‐CDDP cells. The results manifested that miR‐184 expression was repressed in UCA1‐overexpressing Tca8113‐CDDP cells, and miR‐184 expression was elevated in UCA1‐knockdown TSCCA‐CDDP cells (Fig. 3C). All these results suggested that UCA1 could sponge miR‐184 to suppress its expression.

### Downregulation of miR‐184 partly reversed the effect of UCA1 knockdown on proliferation, CDDP susceptibility and apoptosis in CDDP‐resistant OSCC cells

Due to the inhibitory effect of UCA1 on miR‐184 expression, we further assessed whether the function of UCA1 in OSCC development was mediated by miR‐184. Thus, Tca8113‐CDDP and TSCCA‐CDDP cells were transfected with si‐NC, si‐UCA1, si‐UCA1 +  anti‐miR‐NC, or si‐UCA1 +  anti‐miR‐184. XTT assays were applied to estimate cell proliferation and drug resistance. The data indicated that introduction of anti‐miR‐184 partially reversed si‐UCA1‐mediated anti‐proliferation effect on Tca8113‐CDDP and TSCCA‐CDDP cells (Fig. 4A and C). Moreover, si‐UCA1‐induced sensibility of Tca8113‐CDDP and TSCCA‐CDDP cells to CDDP was substantially attenuated following miR‐184 suppression (Fig. 4B and D).

Subsequently, the influence of anti‐miR‐184 on si‐UCA1‐induced apoptosis was also measured. The results implied that UCA1‐knockdown‐induced increase in apoptotic rate (Fig. 4E), caspase‐3 activity (Fig. 4F), Bax protein expression (Fig. 4G) and decrease in BCL2 protein expression (Fig. 4H) were greatly abrogated after miR‐184 downregulation in Tca8113‐CDDP and TSCCA‐CDDP cells. In summary, UCA1 affected OSCC cell growth, apoptosis and CDDP sensitivity by sponging miR‐184.

### SF1 was a direct target of miR‐184

**Figure 5:**
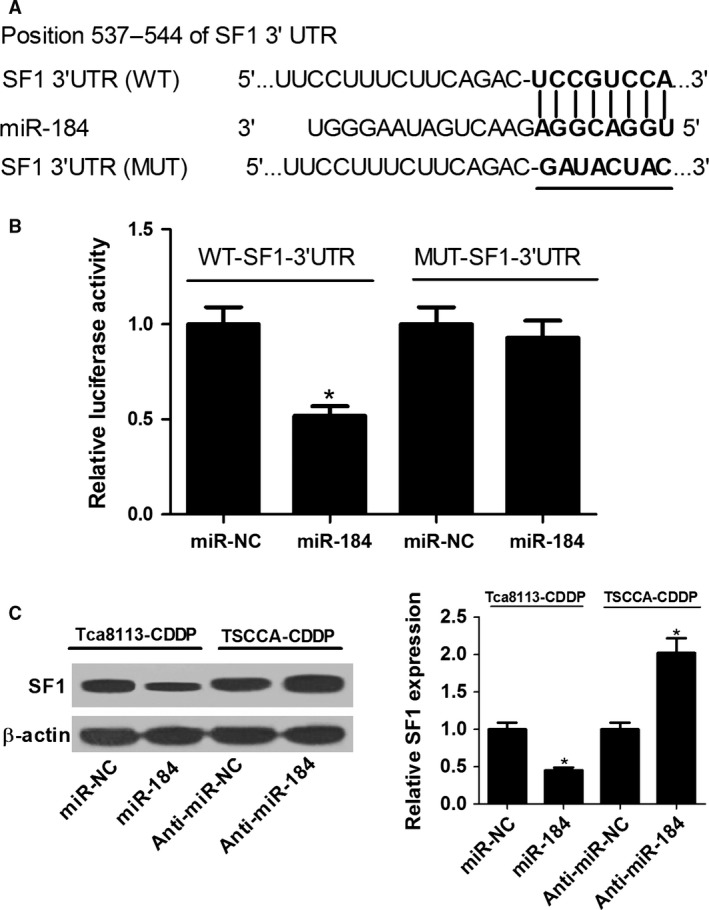
miR‐184 downregulated SF1 expression by binding to the 3′UTR region of SF1 in OSCC cells. (A) Graphical representation of putative binding sites between SF1 3′UTR and miR‐184, as well as mutated binding sequences of SF1‐3′UTR within miR‐184. (B) The WT‐SF1‐3′UTR or MUT‐SF1‐3′UTR reporter were co‐transfected with miR‐NC or miR‐184 mimic into 293T cells to verify the real binding of SF1‐3′UTR and miR‐184 by dual luciferase reporter assays. (C) SF1 protein expression was detected by immunoblotting assays in OSCC cells introduced with miR‐184 mimic or anti‐miR‐184. *P <* *0.05.

In recent years, emerging evidence proposed that lncRNAs could act as ceRNAs to affect the expression of miRNAs and target mRNAs. Accordingly, we aimed to further investigate the mechanism underlying UCA1/miR‐184 axis mediated CDDP resistance in OSCC cells. TargetScan online software demonstrated that SF1 was a candidate target of miR‐184 (Fig. 5A). To further validate the inference, the WT‐SF1‐3′UTR luciferase reporter systems with the putative binding sites and MUT‐SF1‐3′UTR reporter systems with the mutation sites were constructed in 293T cells. The results of luciferase reporter assays showed that compared with miR‐NC group, introduction of miR‐184 mimic tremendously inhibited the luciferase activity of WT‐SF1‐3′UTR reporter systems rather than MUT‐SF1‐3′UTR reporter systems (Fig. 5B). Subsequently, the actual impacts of miR‐184 on SF1 expression were detected in Tca8113‐CDDP and TSCCA‐CDDP cells by immunoblotting assays. As shown in Figure 5C, enforced expression of miR‐184 significantly diminished protein expression of SF1 in Tca8113‐CDDP cells. Conversely, SF1 expression was distinctly improved in anti‐miR‐184‐transfected TSCCA‐CDDP cells. Taken together, miR‐184 downregulated the expression of SF1 by directly targeting the 3′UTR region of SF1 in CDDP‐resistant OSCC cells.

### UCA1 promoted the expression of SF1 by sponging miR‐184 in CDDP‐resistant OSCC cells

**Figure 6:**
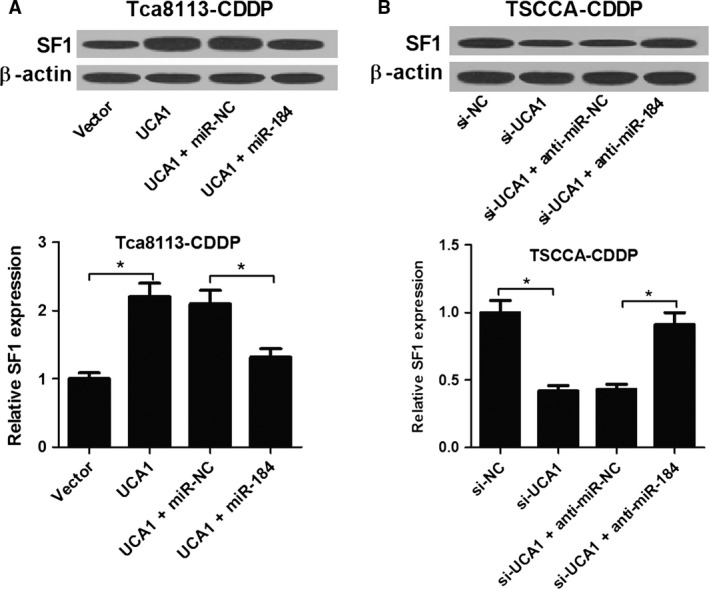
miR‐184 overexpression partially abolished the promotion effect of UCA1 on SF1 expression. (A and B) The protein expression levels of SF1 were determined in Tca8113‐CDDP and TSCCA‐CDDP cells after different treatments by immunoblotting assays. *P <* *0.05.

As described above, UCA1 inhibited the expression of miR‐184 and miR‐184 downregulated the expression of its target gene SF1. Therefore, we attempted to further investigate whether UCA1 could act as a ceRNA to regulate SF1 expression. The protein expression levels of SF1 were firstly assessed in the Tca8113‐CDDP cells transfected with vector, UCA1, UCA1 + miR‐NC, UCA1 + miR‐184. The results indicated that overexpression of UCA1 conspicuously upregulated the expression of SF1, however, restoration of miR‐184 expression abated this effect (Fig. 6A). To further demonstrate the conclusions, TSCCA‐CDDP cells were transfected with either si‐UCA1 alone or in combination with anti‐miR‐184. As might be expected, the reintroduction of anti‐miR‐184 notably overturned si‐UCA1‐elicited inhibition on SF1 expression (Fig. 6B). In general, UCA1 could serve as a ceRNA to upregulate the expression of SF1 via sponging miR‐184.

### UCA1 knockdown enhanced the sensitivity of OSCC cells to CDDP in vivo

**Figure 7:**
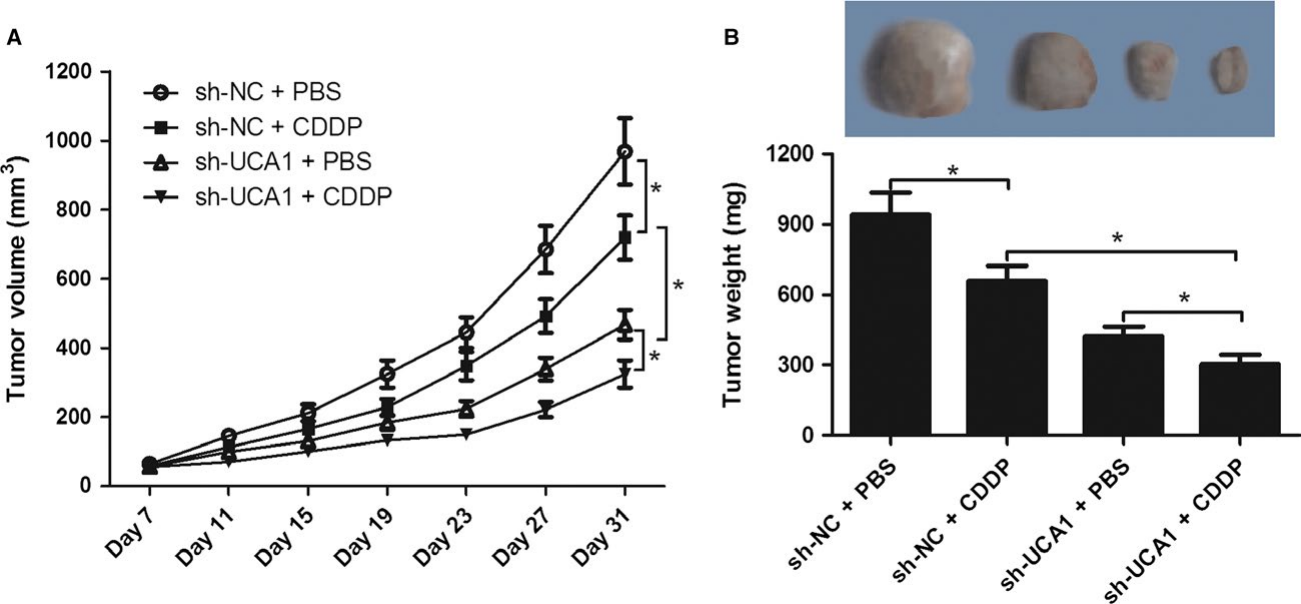
Depletion of UCA1 enhanced CDDP‐mediated tumor inhibition effect in OSCC mice model. Effect of CDDP treatment or UCA1 knockdown on the growth (A) and weight (B) of xenografts derived from Tca8113‐CDDP cells. *P <* *0.05.

In vitro studies results indicated that depletion of UCA1 might have anti‐tumor effect and can enhance CDDP sensibility in CDDP‐resistant OSCC cells. To further validate the conclusion, mouse OSCC transplanted tumor experiments were carried out. We constructed a Tca8113‐CDDP cell line with stable UCA1 deficiency (sh‐UCA1) and a control cell line (sh‐NC). The results manifested that UCA1 knockdown or CDDP treatment notably impeded the tumor growth, exhibited as declined tumor volume (Fig. 7A) and decreased tumor weight (Fig. 7B). Moreover, depletion of UCA1 further aggravated CDDP‐mediated repression on tumor growth. All these data suggested that knockdown of UCA1 blocked tumor growth and increased CDDP sensitivity in OSCC.

## Discussion

Chemoresistance is still a headache for tumor chemotherapy and tremendously hinder therapeutic effect. Recently, emerging studies have focused on exploring the role of lncRNAs in drugs chemoresistance of various cancers including OSCC 15. LncRNA UCA1 is generally regarded as an oncogene, and it is usually highly expressed in a variety of cancers, such as OSCC 10, melanoma 16, gastric cancer 17, and breast cancer 18. Researches also indicated that UCA1 enhanced CDDP resistance in ovarian cancer 19 and bladder cancer 20, and increased 5‐fluorouracil resistance in colorectal cancer 11, indicating the critical role of UCA1 in chemoresistance. CDDP is a widely used chemotherapy drug in OSCC, however, the acquisition of CDDP resistance in OSCC patients markedly restricted its application. Due to the vital effect of UCA1 on the cancer progress and CDDP resistance in OSCC, we intended to further investigate its molecular mechanisms.

In this study, RT‐ qPCR assays revealed that expression levels of UCA1 in OSCC tumor tissues and cells were significantly upregulated compared with normal tissues and cells. Here, we constructed two CDDP‐resistant cells model (Tca8113‐CDDP and TSCCA‐CDDP) to investigate the CDDP resistance mechanisms. The results revealed that the expression levels of UCA1 were upregulated in CDDP‐resistant OSCC cells with respect to corresponding OSCC cells. The data suggested that UCA1 was correlated with CDDP resistance. Further functional analysis indicated that ectopic expression of UCA1 facilitated proliferation and enhanced the chemoresistance of OSCC cells to CDDP. The depletion of UCA1 by si‐UCA1 exhibited reverse results in CDDP‐resistant OSCC cells. To further identify whether si‐UCA1‐induced growth inhibition and CDDP sensitivity was mediated by cell apoptosis. The effect of si‐UCA1 on the apoptosis was also assessed in CDDP‐resistant OSCC cells. The results showed the UCA1 knockdown enhanced the apoptosis rate, caspase‐3 activity, pro‐apoptosis gene Bax expression and suppressed anti‐apoptosis gene BCL2 expression. All these data indicated that UCA1 served as oncogene in OSCC. Consistent with our findings, Yang et al. found that UCA1 silencing repressed proliferation and metastasis, and promoted apoptosis of OSCC cell lines in vitro and in vivo 10. Also, knockdown of UCA1 increased CDDP chemosensitivity in TSCC cells 12.

The bioinformatics predict analysis by the miRcode online website indicated that UCA1 might interact with miR‐184. Reportedly, miR‐184 exerted different functions in various tumors. For instance, miR‐184 acted as tumor repressors and inhibited proliferation and invasion in glioma and breast cancer cells 21, However, miR‐184 also had been identified as oncogenic regulators in hepatocellular carcinoma 22 and TSCC 23. In this context, we revealed that the expression levels of miR‐184 were repressed by UCA1, in accordance with the previous study 24. Moreover, we also demonstrated that downregulation of miR‐184 alleviated si‐UCA1‐mediated CDDP susceptibility and tumor inhibition effect. In other words, miR‐184 might serve as a tumor repressor in CDDP‐resistant OSCC cells and reduced CDDP chemoresistance of CDDP‐resistant OSCC cells.

Recently, accumulating evidence indicated that lncRNAs acted as the ceRNAs to derepress target mRNAs expression by sequestering miRNAs. Consequently, SF1 as a target gene of miR‐184 in OSCC cells was further figured out by the bioinformatics analysis and validated by luciferase reporter assays. SF1, an RNA‐binding protein and also named as zinc finger gene in MEN1 locus (ZFM1), participates in spliceosome assembly of specific pre‐mRNAs 25, 26, 27. Up to now, some literatures have reported the relevance of SF1 and cancers. For instance, the expression levels of SF1 were negatively regulated by the *β*‐catenin/TCF4 complex, which is closely correlated with colorectal carcinogenesis 28. The absence of SF1 could inhibit the tumorigenesis of testicular germ cell tumors 29. Whereas the correlational study of SF1 in cancers including OSCC was deficient, therefore, it is necessary to further investigate its role and regulation mechanism. In this context, we identified that the expression levels of SF1 were regulated by UCA1 and miR‐184 in OSCC cells. The discovery of this regulation pattern was vital and meaningful to further explore the function and molecular mechanism of SF1 in various cancers.

Moreover, we further demonstrated that UCA1 promoted the expression of SF1 and this promotion effect was abated by miR‐184 restoration. These results indicated that UCA1 exert its oncogene role by UCA1/miR‐184/SF1 axis in OSCC cells. In vivo analysis further validated that depletion of UCA1 could boost CDDP‐mediated repression on tumor growth. These findings suggested that suppressing the expression of UCA1 may be a potential therapeutic strategy to effectively enhance therapeutic activity and CDDP susceptibility in OSCC.

In conclusion, we identified that the expression levels of UCA1 were upregulated in OSCC tissue specimens, cell lines, and CDDP‐tolerant OSCC cells. Functional analysis revealed that overexpression of UCA1 promoted proliferation and enhanced resistance to CDDP in OSCC cells. Silence of UCA1 suppressed cell growth and increased CDDP‐induced apoptosis. Molecular mechanisms study further elucidated that UCA1 exerted its function in OSCC cells partly through inhibiting the expression of miR‐184. Moreover, we demonstrated that UCA1 enhanced SF1 expression, while introduction of miR‐184 abated the promotion effect of UCA1 on SF1 expression in OSCC cells. The nude mouse model of xenograft further validated that depletion of UCA1 could enhance CDDP‐mediated repression on OSCC tumor growth. These findings prompt us to better understand function and mechanism of UCA1 in OSCC progression and provide a possible therapy target for OSCC.

## Conflicts of Interest

The authors have no conflict of interest to declare.
